# Extrusion and meniscal mobility evaluation in case of ramp lesion injury: a biomechanical feasibility study by 7T magnetic resonance imaging and digital volume correlation

**DOI:** 10.3389/fbioe.2023.1289290

**Published:** 2024-01-05

**Authors:** M. Severyns, F. Zot, G. Harika-Germaneau, A. Germaneau, G. Herpe, M. Naudin, V. Valle, J. Danion, T. Vendeuvre

**Affiliations:** ^1^ Institut Pprime UPR 3346, Centre National de Recherche Scientifique–Université de Poitiers–ISAE-ENSMA, Poitiers, France; ^2^ Department of Orthopaedic Surgery and Traumatology, Clinique Porte Océane, Les Sables d’Olonne, France; ^3^ Unité de Recherche Clinique Pierre Deniker, CH Henri Laborit, Centre de Recherches sur la Cognition et l’Apprentissage UMR 7295, Centre National de Recherche Scientifique–Université de Poitiers, Poitiers, France; ^4^ CHU de Poitiers, Department of Radiology, LabCom I3M Centre National de Recherche Scientifique–Siemens Healthineers, LMA, UMR CNRS 7348, Université de Poitiers, Poitiers, France; ^5^ CHU de Poitiers, ABS Lab, Poitiers, France; ^6^ CHU de Poitiers, Department of Orthopaedic Surgery and Traumatology, Poitiers, France

**Keywords:** medial meniscus, ramp lesion, digital volume correlation (DVC), magnetic resonace imaging (MRI), displacement fields

## Abstract

**Introduction:** The existing body of literature on the biomechanical implications of ramp lesions is limited, leaving a significant gap in our understanding of how these lesions impact joint kinematics and loading in the medial compartment. This cadaveric biomechanical study aims to address this gap by employing an innovative Digital Volume Correlation (DVC) method, utilizing 7 Tesla Magnetic Resonance Imaging (MRI) images under various loading conditions. The primary objective is to conduct a comprehensive comparison of medial meniscal mobility between native knees and knees affected by grade 4 ramp lesions. By focusing on the intricate dynamics of meniscal mobility and extrusion, this work seeks to contribute valuable insights into the biomechanical consequences of medial meniscus ramp lesions.

**Materials and methods:** An initial set of 7T MRI imaging sessions was conducted on two intact native knees, applying load values up to 1500N. Subsequently, a second series of images was captured on these identical knees, with the same loads applied, following the creation through arthroscopy of medial meniscus ramp lesions. The application of DVC enabled the precise determination of the three components of displacement and spatial variations in the medial menisci, both with and without ramp lesions.

**Results:** The measured directional displacements between native knees and injured knees indicate that, following the application of axial compression load, menisci exhibit increased extrusion and posterior mobility as observed through DVC.

**Discussion:** Injuries associated with Subtype 4 medial meniscus ramp lesions appear to elevate meniscal extrusion and posterior mobility during axial compression in the anterior cruciate ligament of intact knees. Following these preliminary results, we plan to expand our experimental approach to encompass individuals undergoing weight-bearing MRI. This expansion aims to identify meniscocapsular and/or meniscotibial insufficiency or rupture in patients, enabling us to proactively reduce the risk of osteoarthritic progression.

## 1 Introduction

**FIGURE 1:**
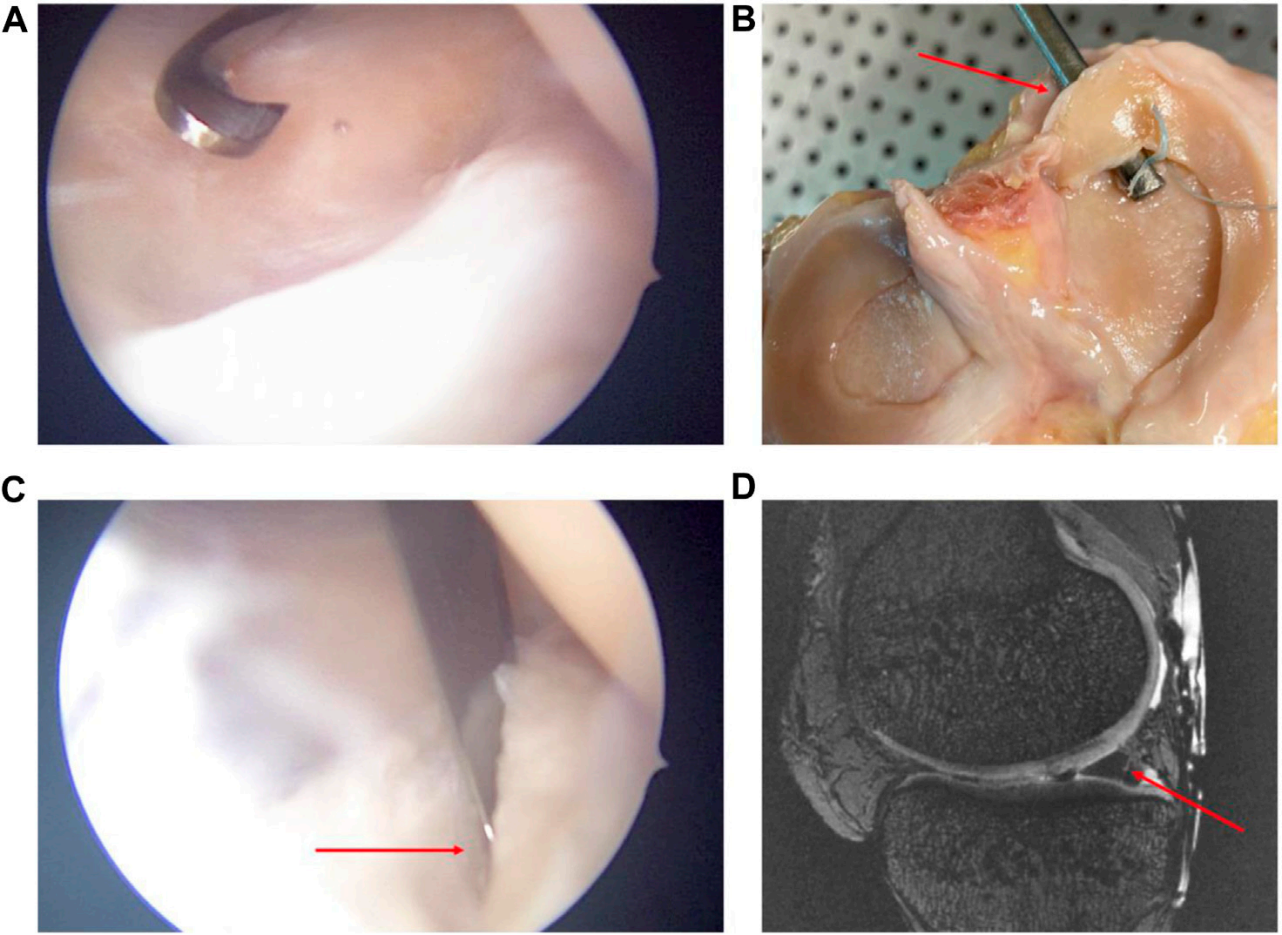
Arthroscopic views **(A,B)** of subtype 4 medial meniscus RL creation by a posteromedial approach. Open view of the knee and its meniscal RL injury after experimentation **(C)**. MRI sagittal view in sequence T2 DESS of the subtype 4 RL injury **(D)**. Red arrows show the subtype 4 RL.

In the context of anterior cruciate ligament (ACL) reconstruction, medial meniscus ramp lesions (RL) and lateral meniscus posterior ramp lesions are prevalent, accounting for over one-third of cases in both primary and revision surgeries (Magosch et al., 2021). Specifically, medial meniscus RLs (Figure 1) are frequently observed traumatic injuries, with a prevalence rate of 21.9% (ranging from 9.0% to 41.7%) at the time of ACL reconstruction (Kunze et al., 2021a; Brophy et al., 2022). They are defined as a distinct category of injuries occurring within the posterior horn of the medial meniscus and its meniscocapsular attachments (Thaunat et al., 2021).

Among the various types of RL, meniscocapsular junction tears, classified as type 1, are the most common, followed by type 4 lesions, which involve a complete tear in the red zone of the meniscus (Thaunat et al., 2021). This underscores the need to understand and address these specific types of meniscal injuries in the context of ACL reconstruction, as they play a crucial role in the overall stability and functionality of the knee joint. Addressing these lesions effectively is essential for optimizing patient outcomes and preventing the long-term complications associated with ACL reconstruction.

At present, understanding of the biomechanical behavior of meniscus is at the forefront of orthopedic discussions (Kunze et al., 2021b; Ollivier et al., 2022). Meniscocapsular and meniscotibial lesions of the posterior horn of the medial meniscus increase anterior tibial translation, internal and external rotation, and pivot shift in ACL-deficient knees (Ahn et al., 2011; Engebretsen et al., 2012; Peltier et al., 2015; Stephen et al., 2016; DePhillipo et al., 2018; Mouton et al., 2020). However, the available literature discussing the biomechanical consequences of RL remains limited. It is not clear whether these lesions affect joint kinematics and loading in the medial compartment (Chahla et al., 2016; Seil, 2018). Recently, Krych et al. (2017) and Mariani et al. (2022) showed that meniscal extrusion was due not only to root lesions but also to meniscotibial ligament (MTL) injuries. Although meniscal extrusion is often the consequence of hyper-pressure in the medial femorotibial compartment, it could also be the cause in different cases, one of them being RL.

Magnetic Resonance Imaging (MRI) plays a pivotal role in investigating meniscal injuries, particularly RL. Its ability to provide detailed, non-invasive, and precise imaging significantly contributes to early detection, appropriate treatment planning, and successful outcomes for patients dealing with this specific meniscal injury. From a biomechanical point of view, Digital Volume Correlation (DVC) is currently used to determine the three components of displacement and spatial variations of a material or structure from volume images (Bay et al., 1999; Buljac et al., 2018; Disney et al., 2018).

The combination of MRI and DVC ensures powerful synergy in medical imaging and biomechanical analysis. While MRI provides detailed and high-resolution images of internal structures, capturing intricate anatomical information, DVC is a sophisticated image analysis technique used to quantify deformation and strain in complex structures. The integration of MRI with DVC enhances biomechanical studies by correlating structural information with mechanical behavior. This correlation is invaluable in fields such as orthopedics, where understanding the mechanical properties of tissues is crucial for the development of effective treatment strategies and injury prevention programs.

This biomechanical work concerned a case study, of which the objective was to compare medial meniscal mobility between native knees and knees with grade 4 RL by a DVC method using 7 Tesla MRI at different loadings. The hypothesis of this study is that medial meniscus RL increases meniscal mobility and extrusion.

## 2 Materials and methods

### 2.1 Specimen preparation and loading device

**FIGURE 2:**
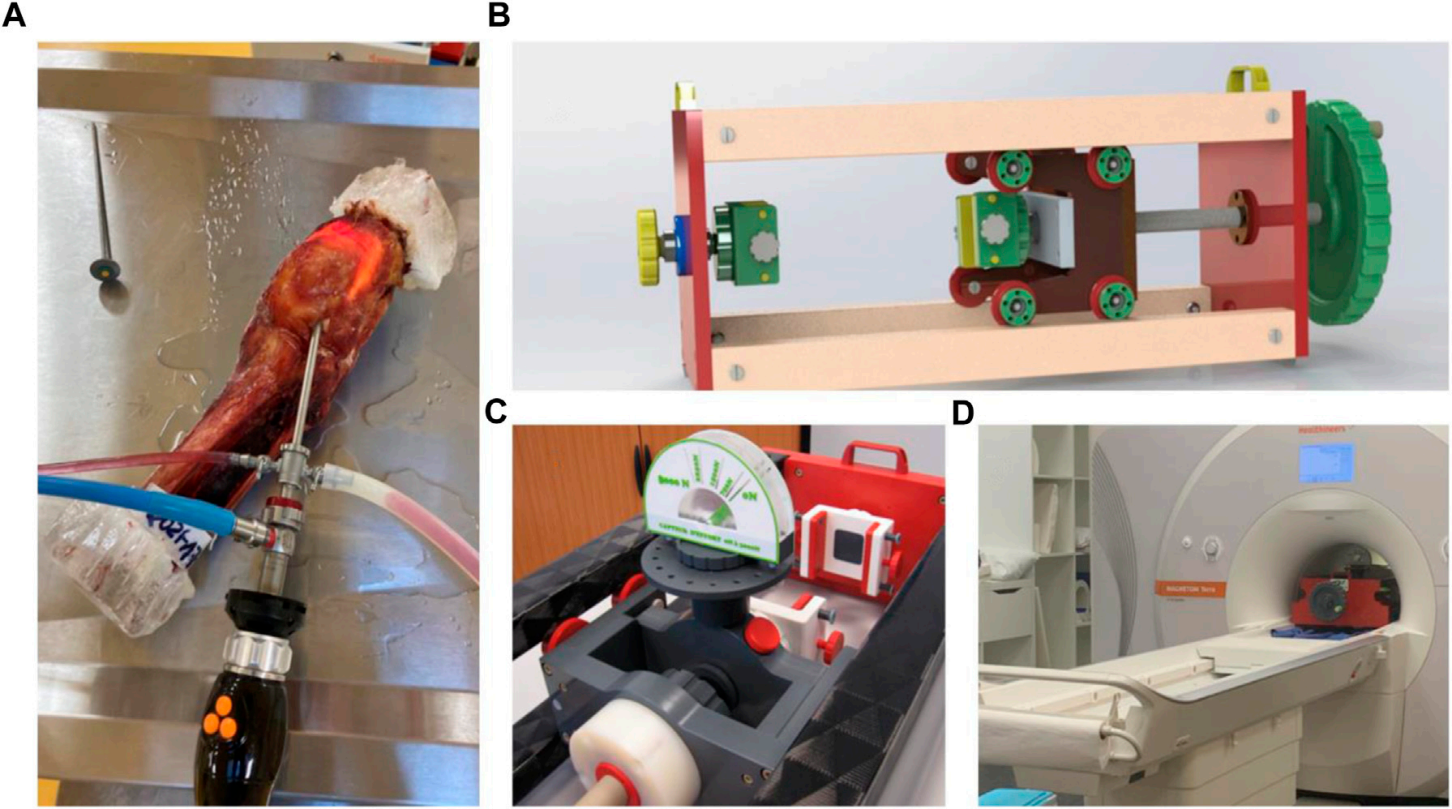
Anatomical view of a knee segment with its proximal and distal fixations of rigid polyurethane resin **(A)**. MRI-compatible loading bench, **(B)** pressure sensor, **(C)** *in situ* loading setup in the 7T MRI device **(D)**.

This experimental study was conducted on two cadaveric knees that had a Normo-axial morphotype with a meniscal (and cartilaginous state deemed intact during the 7 Tesla MRI (7T MRI) examination. Specimen preparations were carried out at the Anatomy Laboratory of the University of Poitiers (DC-2019-3704 Université de Poitiers). The epidemiological data showed a 63-year-old man (73 kg, named Knee 1) and an 81-year-old woman (79 kg, named Knee 2) with no osteoarticular history and whose CT scan measurements of HKA angle were 178.9° (leg length: 123.4 cm) and 177.8° (leg length: 127.7 cm) respectively. The knees were then disarticulated below the hip and above the ankle and dissected, preserving the entire capsule and peripheral ligaments without opening the knee joint. Proximal and distal fixations of rigid polyurethane resin were made in order to facilitate fixation of the anatomical parts on an MRI-compatible loading bench without ferromagnetic components and designed specifically for this series of experiments (Figure 2). The knee was positioned in the loading device in the sagittal direction, aligning the loading axis with the orientation of the MRI tube. The loading bench could impose compression loading up to 1500N. Imposed load ability was preliminarily calibrated and controlled during experiments by a specific homemade hydraulic sensor. Biomechanical experiments were conducted in accordance with established guidelines for *in vitro* studies (Wilke et al., 1998). Cadaveric knees from fresh and non-formalin-fixed specimens were cryopreserved after the primary dissection phase. Before each experiment, the thawing protocol consisted of placing the cadaveric segments at room temperature for 48 h in order to optimize the elasticity/stiffness relationship and to get closer to the physiological conditions found in living patients.

### 2.2 Experimental protocol

Images were acquired using a 7-Tesla scanner (Magnetom Terra, Siemens Healthineers, Erlangen, Germany) with a dedicated Transmit/Receive 28-channel knee coil (QED, Mayfield Village, OH, United States). Anatomical images were acquired using 3D T2 DESS 0.35 mm isotropic (TR 8ms, TE 2.48 ms, slice 0.35 mm, 464 × 464 matrix size, FA 24°, FOVr 164mm, FOVp 72.4%, 1 Nex, PAT 2). An initial series of 7T MRI imaging sessions were conducted on native knees under axial loads of 750N and 1500N, equivalent to one to two times the body weight load. Subsequently, a second set of images was captured on the same knees, employing the same loads, following the creation of grade 4 medial meniscus RL (Thaunat et al., 2021) using a posteromedial instrumental approach under arthroscopy (Figure 1).

### 2.3 Digital volume correlation and assessment criteria

To measure displacement fields through DVC, a sub-volume of voxels (D) is defined at each voxel in the initial image. The position of each sub-volume is subsequently determined by assessing the degree of similarity within the initial image. To achieve this objective, a correlation sub-volume is characterized by the voxel values denoted as $f\left(\vec{X}\right)$ at the reference state, with $\vec{X}$ representing the initial position vector. The position of the desired sub-volume in the deformed state is designated as $\vec{x}$ , and the corresponding grey levels are denoted as $g\left(\vec{x}\right).$ The degree of similarity between a sub-volume in the initial and deformed states is assessed through a correlation coefficient (Germaneau et al., 2007) established by optimizing a functional $f\left(\vec{X}\right)-g\left(\vec{\phi }\left(\vec{X}\right)\right)$ , where $\vec{\phi }$ signifies the material transformation between the deformation states. Tricubic interpolation of grey levels was employed to attain displacement field measurements with subvoxel resolution. This non-contact method facilitates the measurement of volume displacements within the structure, ranging from 1 µm to several tens of millimeters (Germaneau et al., 2008; Valle et al., 2019).

The overlay of constrained and unconstrained MRI images was carried out through semi-automated tibial registration of the image sequences using 3DSlicer software (Version 4.11, Kitware, France). Manual segmentation of the medial meniscus was performed by an experienced surgeon with expertise in medical image analysis, utilizing the first MRI scan to extract the relevant meniscal area. This singular segmentation aimed to delineate the specific region of interest to be analyzed through the DVC process. Given the stationary nature of the knee bones, meticulous image registrations were conducted between steps, referencing the bone structures. This approach ensured precise alignment, facilitating accurate measurements in the DVC computation area associated with the meniscus.

**FIGURE 3:**
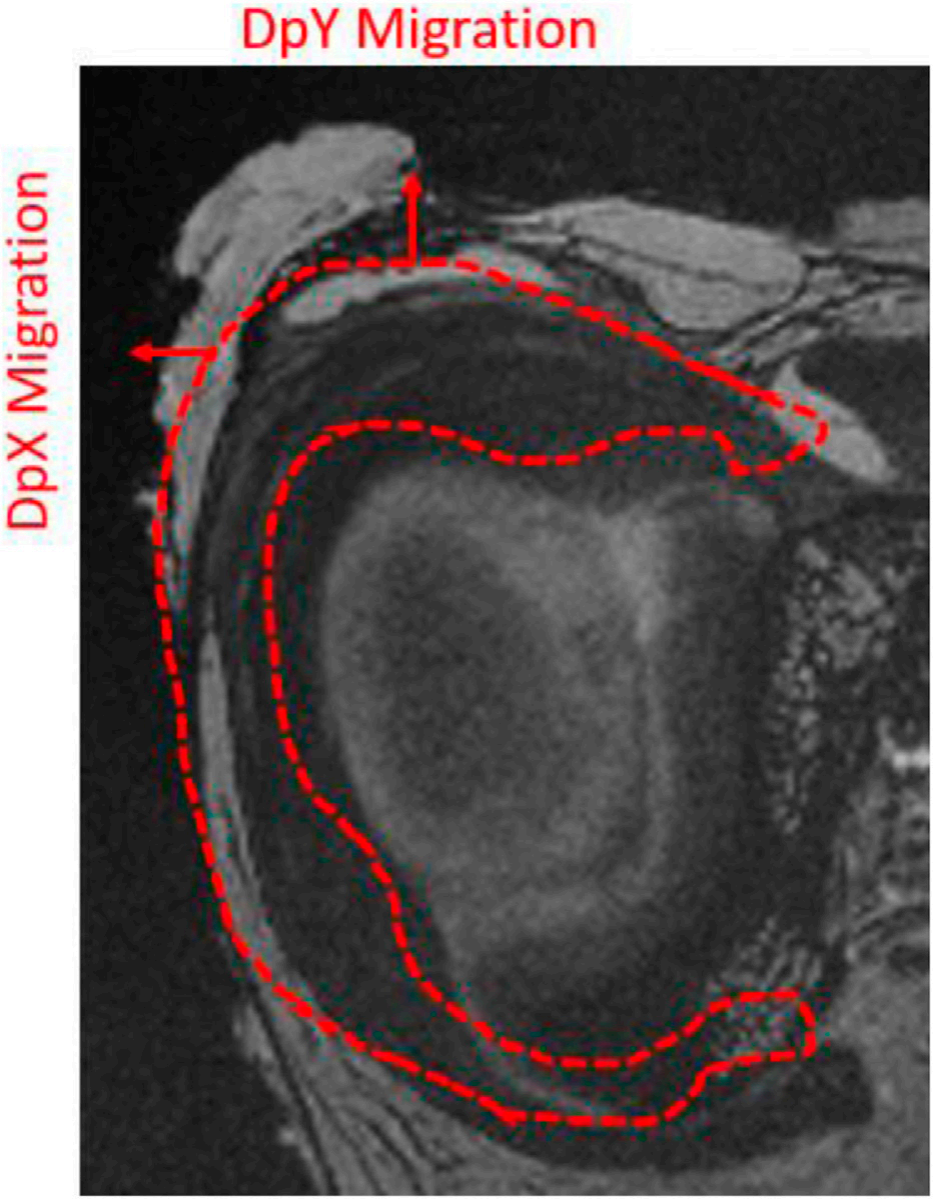
Illustration of the orientation of the displacement fields analyzed in DVC on a coronal MRI slice of a right cadaveric knee.

The displacement fields were analyzed in three dimensions. Observation of displacements along the *X* and *Y* directions facilitated analysis of anteroposterior and lateromedial migration of the meniscus after axial compression, with the knee positioned in full extension (Figure 3). The measurement uncertainty provided by the DVC process for these MRI data was assessed at 0.05 voxel.

## 3 Results

**FIGURE 4:**
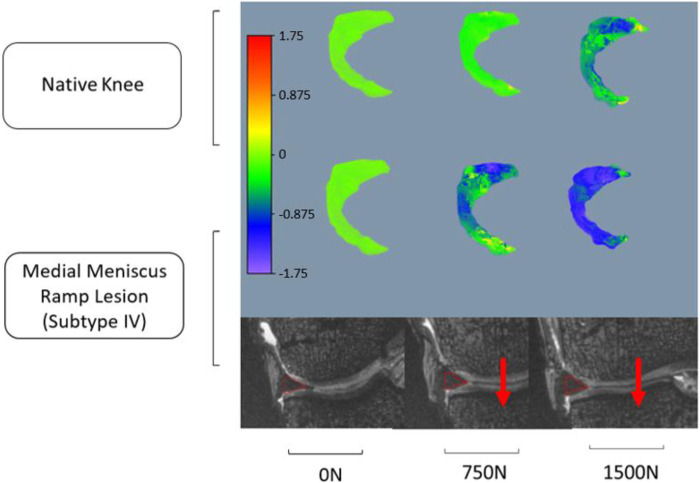
Observation of the displacement fields (in mm) after DVC assessment for knee 1 according to the lateromedial direction (X).

**FIGURE 5:**
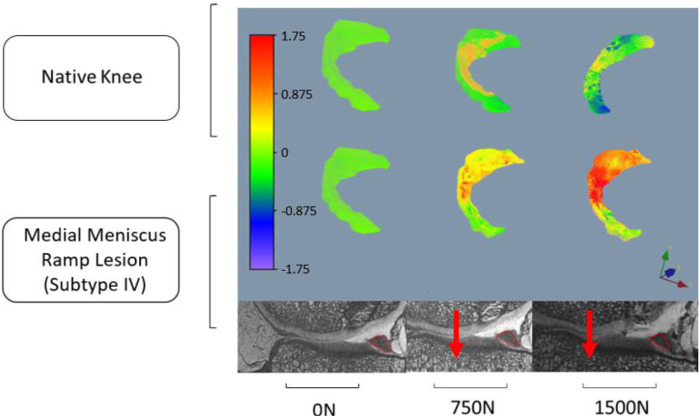
Observation of the displacement fields (in mm) after DVC assessment for knee 1 according to the anteroposterior direction (Y).

**FIGURE 6:**
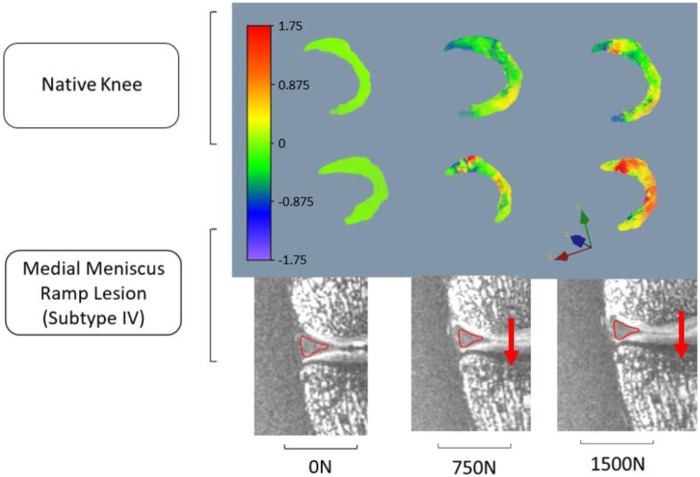
Observation of the displacement fields (in mm) after DVC assessment for knee 2 according to the lateromedial direction (X).

**FIGURE 7:**
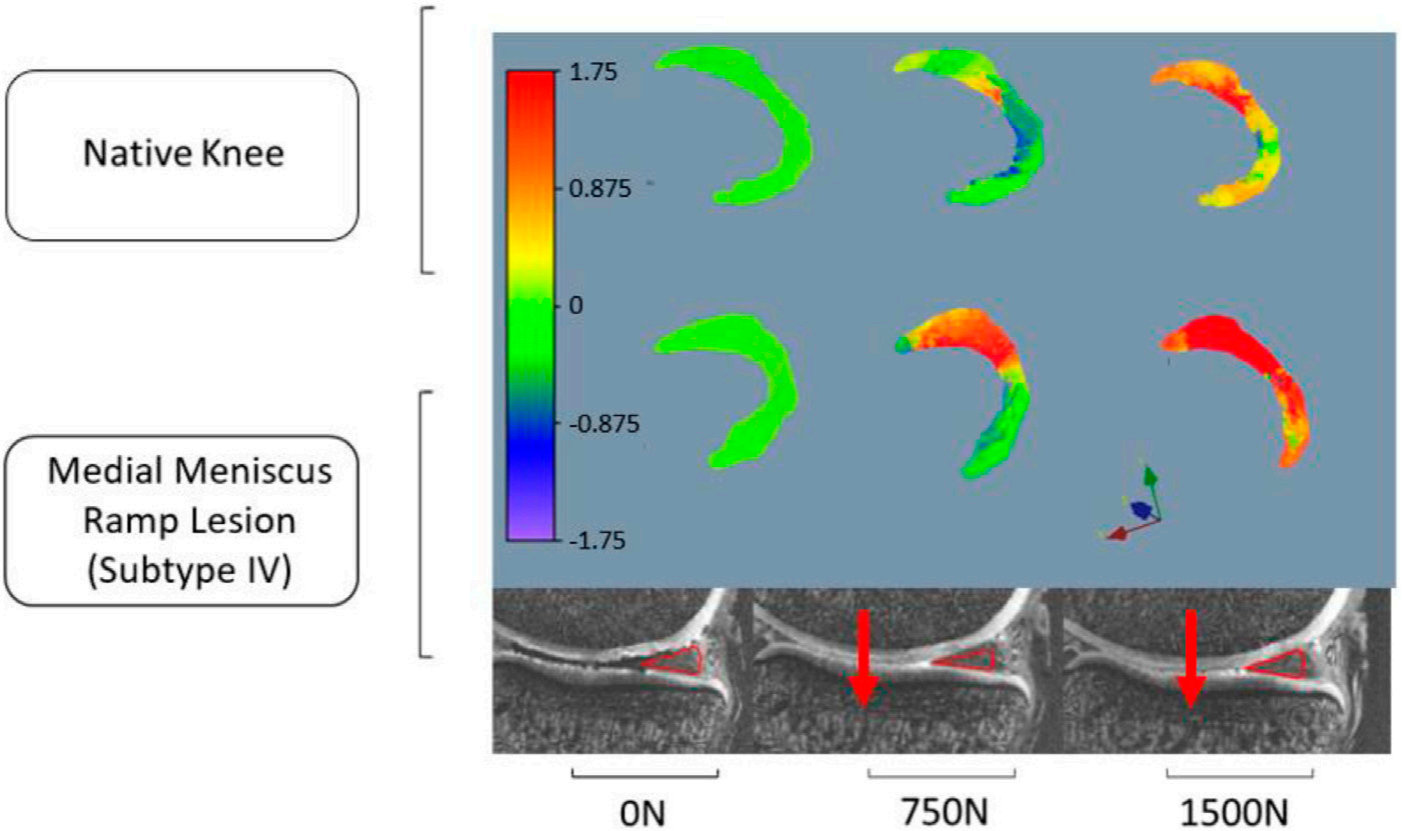
Observation of the displacement fields (in mm) after DVC assessment for knee 2 according to the anteroposterior direction (Y).

The red contour in Figure 3 illustrates meniscus migration during loading. The directional displacements of the medial meniscus were measured by DVC. Figures 4–7 show the directional displacement fields obtained by DVC after the application of 0N, 750N, and 1500N of axial load.

Mean displacements were measured on the posterior segment of the medial meniscus, anteriorly to the RL. In the lateromedial direction (X), the mean displacements measured at 1500N load were −0.983 mm (±0.027 mm) on the knee 1 *versus* −1.568 mm (±1.097 mm) on the same knee with RL injury. For knee 2, the mean displacements measured according to X with the same load were 0.738 mm (±0.133 mm) before and 1.647 mm (±0.031 mm) after RL injury. Positive or negative values are associated with the knee’s laterality. With a grade 4 medial meniscal RL, the meniscus increases its extrusion in the frontal plane (Figures 4, 6).

In the anteroposterior direction (Y), the mean displacements measured at 1500N load were 0.167 mm (±0.183 mm) on knee 1 *versus* 1.150 mm (±0.287 mm) on the same knee with RL injury. For knee 2, the mean displacements measured in Y with the same load were 0.739 mm (±0.132 mm) before and 2.559 mm (±0.459 mm) after RL injury. With grade 4 RL, the posterior segment of the medial meniscus increases its posterior extrusion in the sagittal plane (Figures 5, 7).

All data on the anteroposterior and/or lateromedial migration of the meniscus after axial compression between native knees and injured knees are summarized in Table 1.

**TABLE 1:** Displacement measurement obtained by DVC after application of 0N, 750N, and 1500N axial loads between native knees and injured knees (in mm).

Displacement measurement (mm)	KNEE 1							
	Native Knee			Medial Meniscus RL				
	0N	750N	1500N	0N		750N	1500N	
Lateromedial direction (DpX)								
Mean	−0.046	−0.281	−0.983	0.016		−0.683	−1.568	
Min-Max	−0.077–0.006	−0.407–0.121	−1.599–0.008	−0.031–0.045		−2.041–0.301	−3.154–0.412	
SD	0.015	0.100	0.272	0.019		0.346	1.097	
Anteroposterior direction (DpY)								
Mean	−0.075	0.141	0.167	−0.029		0.318	1.150	
Min-Max	−0.094–0.005	0.047–0.221	−1.014–0.691	−0.056–0.018		0.104–0.735	1.091–1.732	
SD	0.007	0.041	0.183	−0.029		0.105	0.287	

	KNEE 2							
	Native Knee			Medial Meniscus RL				
	0N	750N	1500N	0N	750N			1500N
Lateromedial direction (DpX)								
Mean	0.018	0.604	0.738	0.014	0.978			1.647
Min-Max	−0.026–0.041	0.365–0.967	0.487–0.774	−0.032–0.045	0.13615–2.406			1.101–2.627
SD	0.014	0.137	0.133	0.020	0.505			0.309
Anteroposterior direction (DpY)								
Mean	−0.029	0.010	0.739	−0.028	1.746			2.559
Min-Max	−0.054–0.013	−0.483–0.249	0.480–0.981	−0.052–0.012	0.821–2.582			1.472–3.166
SD	0.010	0.073	0.132	0.013	0.364			0.459

## 4 Discussion

The main finding of this study is that medial meniscus RL injury increases meniscal extrusion, which is defined as the internal displacement of the medial meniscus with regard to the medial margin of the tibial plateau, and meniscal posterior mobility during axial compression. This biomechanical study confirms the hypothesis that RL could be responsible for meniscal extrusion usually considered as the result of meniscal root lesion or disruption of the coronary ligaments or isolated MTL injury (Chahla et al., 2016; Krych et al., 2017; Naendrup et al., 2019; Thaunat et al., 2021; Mariani et al., 2022).

Meniscal extrusion is recognized as a significant predictor of accelerated joint degeneration (Hein et al., 2011; Krych et al., 2017). Diagnosing meniscal extrusion is crucial, due not only to the acute functional limitations it imposes on patients but also because of its direct association with osteoarthritis (OA), a condition often observed in the elderly (Sugita et al., 2001; Guermazi et al., 2015). Meniscal extrusion has been shown to diminish the hoop function of the meniscus, increasing the risk of knee OA (Ozeki et al., 2022). In our case, RLs are prevalent meniscal injuries, typically occurring in cases of ACL rupture or knee laxity associated with anterior cruciate ligament insufficiency. These injuries are particularly common in patients under 30 years of age and male patients (Liu et al., 2011). Current literature consensus indicates that meniscal extrusion, whether with or without ACL deficiency, amplifies mechanical loading. Consequently, abnormal mechanical stress can trigger a pathological response in joint tissues, leading to cartilage degradation characteristic of knee OA, particularly in the medial compartment (Daszkiewicz and Łuczkiewicz, 2021). Similarly, a significant correlation has been established between the degree of medial meniscus extrusion and the onset of post-arthroscopic osteonecrosis of the knee (Yamaguchi et al., 2021).

Naendrup et al. (2019) found non-significant results on knee medial stress due to RL injuries. No differences were found with respect to kinematics, *in situ* forces in the ACL, or bony contact forces between intact knees and knees with a ramp lesion. However, this study was performed on ACL-intact knees with a maximum axial compression load of 200N on a freedom robotic testing system. While the same authors think that the indications for RL repair may be limited, there are a number of reasons that seem to justify meniscal repair in addition to the risk of OA degeneration due to RL. Medial menisci with RL are less stable and could progress toward a bucket-handle tear, especially in subtype 4 or 5 lesions (Thaunat et al., 2021). In addition, DePhilippo et al. (2018) have observed that meniscotibial and meniscocapsular lesions of the posterior segment of the medial meniscus increase knee anterior tibial translation, internal and external rotation, and pivot shift in ACL-deficient knees. Optimal treatment has been debated in the literature, especially for stable RL, although good outcomes have been shown both with and without repair (Brophy et al., 2022). Healing rates of RL were significantly better when lesions were repaired and surgical procedures appeared reliable (Hatayama et al., 2020). Recently, Park et al. (2022) analyzed the joint capsule adjacent to the medial meniscus and found that the perimeniscal joint capsule had collagen fiber orientation similar to that of circumferential meniscal fibers, potentially playing a role in preventing extrusion. They also found that the circumferential rim augmentation suture reduced the degree of meniscal extrusion while restoring meniscal function, potentially preventing the progression of arthritis in a rabbit root tear model and by means of porcine knee biomechanical analysis (Park et al., 2022).

The typical method for quantifying extrusion involves measuring the distance between the medial edge of the tibial plateau and the most prominent medial point of the medial meniscus (Choi et al., 2010). Existing literature suggests that meniscal extrusion exceeding 3–4 mm can have biomechanical implications on the tibiofemoral compartment contact area and pressures (Debieux et al., 2021). It is important to note that our findings, although lower than these thresholds, pertain to knees without osteoarthritis (OA) and ACL insufficiency.

Furthermore, even in healthy knees, some studies have demonstrated meniscal mobility. In an ultrasound-based study, Kawaguchi et al. (2012) showed that physiologic loading causes mild meniscal extrusion. Another ultrasound study demonstrated that the posterior portion experiences greater extrusion than the anterior portion, particularly as regards the medial meniscus (Sharafat Vaziri et al., 2022). Additionally, research has explored meniscal displacements and 3D morphological changes under knee weight-bearing and early flexion conditions in healthy adults, utilizing MRI (Liu et al., 2021). Of note, data through MRI regarding injured knees with volume quantification are lacking. Our study stands out as the first to evaluate meniscal displacement using DVC under various axial compression loads. The direct correlation DVC technique is reliable and reproducible, provided that study sub-volumes are optimized (Palanca et al., 2015; Marter et al., 2020).

The study has some limitations. First, it was a cadaver study. Although we tried to optimize the elasticity/stiffness ratio with respect to ethical use and institutional thawing procedure, the performance of this study on fresh cadaver knees would have allowed us to come even closer to reproducing the physiological conditions of meniscal displacement in living patients.

Secondly, the study was conducted using only two anatomical specimens. The results underscored the specific observations associated with each specimen. Mediolateral extrusion was comparable between the two knees, while variability was noted in the anteroposterior direction. In the instance of one knee (knee 2), native anteroposterior mobility surpassed that of the other. The results indicated that mobility after RL could be proportionate to mobility in the intact condition. This work served as a case study and proof of concept, demonstrating the integration of 7T MRI and DVC to characterize the mechanical behavior of the meniscus in knee articulation under loading. While intriguing initial conclusions were drawn, it is essential to validate these results through additional experiments involving a larger number of specimens.

This study was conducted with the knee in extension because of the 7T MRI device. However, extrusion of the meniscus’s medial body seemed to be greater in full extension compared to any other flexing angles. Mechanical loading can significantly deform the menisci in knee extension; however, this effect is limited during knee flexion (Liu et al., 2021). In contrast, anteroposterior mobility commonly increases with the rise of knee flexion motion (Sharafat Vaziri et al., 2022). For this reason, the data concerning lateromedial displacement (X) appear to be more informative.

It is possible that variability between our results and the findings of future studies could be due to the characteristics of the created lesions. The definition of RL is constantly debated, especially in terms of length. RL has been commonly defined as a 25 mm tear (Chahla et al., 2016; Balazs et al., 2019). However, during our experimentation, the RL length we were able to achieve was 20 and 21 mm in accordance with DePhillipo et al. (2018), who clearly established that the length of the posteromedial meniscocapsular junction may not exceed 20 mm because otherwise it would be extended to the midportion of the meniscus.

Utilization of a 7T MRI device brought forth numerous advantages and advancements. 7T MRI offers spatial resolution superior to 3T MRI, enabling the capture of highly detailed images of the knee joint and meniscus. This heightened clarity proves invaluable when evaluating intricate structures such as meniscal root attachments and subtle pathological changes within the meniscus. It bears mentioning that the biomechanical research presented in this study could be designed for routine use on the 1.5T or 3T scanners commonly employed in clinical practice. Subsequent studies need to be conducted to assess the capacity of 1.5T or 3T scanners to perform DVC measurements of mechanical fields within the meniscus with adequate resolution.

## 5 Conclusion

In this work, original experiments were developed to perform *in situ* mechanical loadings of anatomical knee specimens in a 7T MRI device and to measure volume displacements in intact meniscus or after lesion. Subtype 4 medial meniscus RL injury increases meniscal extrusion and meniscal posterior mobility during axial compression in ACL-intact knees. Indeed, the meniscotibial ligament and meniscocapsular junction seem to behave like the belt and suspenders of the medial meniscus. Although these data improve biomechanical knowledge of RL, their clinical impact in ACL-intact knees must be evaluated in the long term before proposing systematic arthroscopic repair. Following the proof of concept developed in this work, the plan is to extend the experimental approach to encompass individuals undergoing weight-bearing MRI. This expansion aims to measure *in vivo* deformation in soft structures, identifying meniscocapsular and/or meniscotibial insufficiency or rupture in patients.

## Data Availability

The raw data supporting the conclusions of this article will be made available by the authors, without undue reservation.
